# Sex-related differences in the physical, functional, physiological, and emotional profile of adults with chronic vestibular hypofunction: a cross-sectional EXERVEST study

**DOI:** 10.3389/fresc.2026.1890080

**Published:** 2026-08-26

**Authors:** Maitane Ruiz-Rios, Asier Lekue-Madinabeitia, Mikel Tous-Espelosin, Pablo Corres, Ibai Garcia-Tabar, Lucas Tojal-Sierra, Antonio Moreno-Rueda, Sara Maldonado-Martin

**Affiliations:** 1 GIzartea, Kirola eta Ariketa Fisikoa Ikerkuntza Taldea (GIKAFIT), Society, Sports, and Physical Exercise Research Group, Department of Physical Education and Sport, Faculty of Education and Sport-Physical Activity and Sport Sciences Section, University of the Basque Country (EHU), Vitoria-Gasteiz, Basque Country, Spain; 2 Physical Activity, Exercise, and Health Research Group, Bioaraba Health Research Institute, Vitoria-Gasteiz, Basque Country, Spain; 3 Otorrinolaringology Services of OSI Araba-Osakidetza Basque Health Service, Araba University Hospital, Vitoria-Gasteiz, Basque Country, Spain; 4 Epidemiology and Public Health Group, Bioaraba Health Research Institute, Vitoria-Gasteoz, Basque Country, Spain; 5 Cardiology Services of OSI Araba-Osakidetza Basque Health Service, Araba University Hospital, Vitoria-Gasteiz, Basque Country, Spain; 6 Medicine Department. University of the Basque Country (EHU), Vitoria-Gasteiz, Basque Country, Spain

**Keywords:** cardiorespirarory fitness, postural control, sex diferences, sleep quality, vestibular hypofunction

## Abstract

**Background:**

Chronic vestibular hypofunction (CVH), often resulting from illness or injury, is linked to sedentary behaviour, poor sleep, and comorbidities. This cross-sectional descriptive study characterized the physical, functional, physiological, and emotional profiles of adults with CVH and explored sex-related differences within this population.

**Methods:**

Participants (*N* = 67, 58 yrs, 43 women) with CVH were assessed on their body composition, cardiorespiratory fitness (CRF), balance and fall risk (posturography and Modified Dynamic Gait Index, MDGI), health-related quality of life (Dizziness Handicap Inventory, DHI), emotional profile (Beck Depression Inventory [BDI-II] and Beck Anxiety Inventory [BAI]), level of physical activity (PA), sedentary behaviour, and sleep quality (accelerometry).

**Results:**

As a group, participants showed a waist-to-hip ratio of 0.8 (0.7–0.9), mean MDGI score of 61.0 points, standard somatosensory and visual data from posturography, a median values of DHI, BDI-II, and BAI of 26.0 (16.0–34.0), 11.0 (4.0–17.5), and 12 (7.8–20.3), respectively; sleep efficiency >85%, greater sedentary behaviour (∼54%) relative to PA levels, reduced total sleep time (TST) and a higher frequency of nocturnal awakenings than recommended (35.7 ± 14.7 min). Sex-related differences were observed (*P* < 0.05): women were older, showed higher fat mass (+18%), better sleep efficiency (+2.4%), more bedtime (+5.7%), and TST (+8.0%), but lower fat-free mass (−9.7%), visual dependence (−18%), and CRF (−20.7%) compared to men. No sex differences were observed in dizziness handicap, anxiety, depression, or PA levels.

**Conclusions:**

Adults with CVH exhibited a multidimensional clinical profile with sex-related differences in several physical, functional, and physiological outcomes. These findings support comprehensive clinical assessment and warrant longitudinal studies to determine the clinical relevance of these differences for personalized programs.

## 1 Introduction

Chronic vestibular hypofunction (CVH) is a common disorder of the peripheral vestibular system that affects more than 5% of adults and over 25% of older adults (1). It may result from unilateral or bilateral loss of vestibular function associated with several underlying disorders, including vestibular neuritis, Ménière's disease, ototoxicity, vestibular schwannoma, or age-related vestibular degeneration, although approximately half of the cases remain idiopathic (1, 2). Acute vestibular asymmetry may cause vertigo, nausea, and spontaneous nystagmus, while chronic manifestations include gait instability, oscillopsia, head movement-induced symptoms, spatial disorientation, and impaired navigation (3). Consequently, CVH negatively affects daily function and health-related quality of life (HRQoL), increasing fall risk, disability, anxiety, and depression (1, 3).

Beyond its well-established effects on balance, gaze stability, and mobility, CVH is increasingly recognized as a complex disorder with consequences across multiple dimensions of health and functioning. Thus, anxiety, depressive symptoms, and reduced HRQoL are frequently reported among people with CVH, highlighting the complex interactions between vestibular dysfunction, emotional wellbeing, and functional disability (1, 4, 5). Chronic vestibular dysfunction has also been associated with sleep disturbances, with growing evidence suggesting a bidirectional relationship in which dizziness may disrupt sleep, while poor sleep may exacerbate vestibular symptoms and impair central compensation (6–9). To minimize symptom provocation, many individuals reduce physical activity (PA), resulting in greater sedentary behaviour, lower adherence to recommended exercise levels, reduced cardiorespiratory fitness (CRF), and an increased risk of cardiometabolic comorbidities (3, 10, 11). Because regular PA and CRF are inversely associated with chronic disease risk (10), assessing PA and CRF in this population may help identify risk of falls, frailty, and cardiometabolic disease, and may support monitoring of treatment progress (11).

Accumulating evidence also suggests that biological sex may influence vestibular function and disease susceptibility, and therefore, a higher prevalence among women than men has been observed (3, 10, 11). Several mechanisms have been proposed to explain sex-related differences in CVH, although direct evidence in humans remains limited. Gonadal hormones, particularly oestrogen and progesterone, modulate inner-ear fluid regulation and otoconial metabolism, and their decline around the menopausal transition has been associated with a higher prevalence of vestibular symptoms and with specific conditions such as benign paroxysmal positional vertigo and Ménière's disease (12, 13). Anatomical and functional sexual dimorphism of the peripheral and central vestibular structures has also been proposed as a contributing mechanism (14). Despite this body of work, recent reviews concluded that sex- and gender-related evidence in the audio-vestibular field remains relatively limited and fragmented across single outcomes or single conditions (12, 15). Previous studies have primarily focused on isolated aspects of CVH, such as balance performance, PA, psychological symptoms, or HRQoL (3, 11, 16), or have explored associations between PA, anxiety, handicap, and balance in chronic dizziness (16). However, comprehensive evaluations integrating physical, functional, physiological, behavioral, and emotional outcomes within the same cohort are scarce. Moreover, direct comparisons between women and men across these multiple health domains remain limited, making it difficult to determine whether clinically relevant sex-related differences exist that could influence patient assessment or future adjuvant strategies.

Therefore, the present cross-sectional, descriptive study aimed to comprehensively characterize the physical, functional, physiological, and emotional profile of adults with CVH, with particular emphasis on sex-related differences. Specifically, we assessed body composition, CRF, balance performance, HRQoL, anxiety, depression, PA, sedentary behaviour, and objectively measured sleep quality. We hypothesized that women and men with CVH would exhibit distinct multidimensional profiles; however, this cross-sectional design does not allow for causal mechanisms underlying any observed differences to be established.

## 2 Material and methods

This study presents a cross-sectional descriptive analysis of baseline data from the EXERVEST study. Published normative values were included solely to facilitate the clinical interpretation of selected outcomes and were neither considered a contemporaneous control group nor used for inferential statistical comparisons. To minimize inter-rater variability, all clinical assessments were performed by the same trained investigator using standardized protocols.

### 2.1 Participants

All participants (*N* = 67, 58.0 yrs [interquartile range (IQR) = 52.0–66.0], 64.2% women) had a diagnosis of peripheral unilateral or bilateral CVH, with condition duration ranging from one to 44 years (Supplementary File 1). All recruited patients were being followed in a “vestibular disorder” clinic and presented, among other symptoms, with unsteadiness lasting more than six months. All participants underwent a video head-impulse test, showing unilateral or bilateral gain values below 0.8 in the horizontal semicircular canal assessment, along with saccades. In a few specific cases within the Ménière's disease group, CVH was confirmed through caloric testing, showing asymmetry greater than or equal to 25%. The diagnoses included: 1) Ménière's disease (16.4%); 2) vestibular neuronitis (20.9%); 3) labyrinthitis (13.4%); 4) tumour of the internal auditory canal (19.4%; e.g., neurinoma and meningioma); 5) bilateral CVH (7.5%; e.g., ototoxicity, Lyme disease, and polyneuropathy); and 6) unknown (22.4%). Participants were excluded if they had neurological or neurodegenerative pathologies, sensory or motor impairments that hindered exercise performance, or serious medical conditions, such as uncontrolled hypertension or severe psychiatric/musculoskeletal diseases, that could compromise their safety during the exercise program. All other inclusion and exclusion criteria have been specified in the study protocol (17). Although heterogeneity in etiologies was considered during data collection, the analysis focused on overall trends in clinical improvement, which is appropriate given the variability in diagnostic certainty and progression across subgroups. The study was approved by the Research Ethics Committee of the OSI ARABA (Certificate No. 2021-095, January 19, 2023, second version), and written informed consent was obtained from all participants. The study has been registered on ClinicalTrials.gov under ID NCT05192564 (2022, January 8th).

### 2.2 Measurements

Age, smoking status, diabetes mellitus, and medication use were self-reported. Anthropometric assessments included stature, body mass, waist and hip circumferences, waist-to-hip ratio (WHR), fat-free mass (FFM), and fat mass (FM). Resting electrocardiography and 24-h ambulatory monitoring assessed systolic blood pressure (SBP), diastolic blood pressure (DBP), and heart rate.

Balance and fall risk were measured using the Modified Dynamic Gait Index (MDGI), which involved eight timed walking tasks (over a distance of 6.1 m) scored for completion time, assistance level (0–2) and gait pattern (0–3) for a maximum score of 64 (18). Participants’ equilibrium and instability were also assessed using a dynamic posturography test (Synapsys Posturography System, Synapsys, Marseille, France) (19) incorporating the Sensory Organisation Test (SOT). The SOT evaluates postural control and visual dependence across six sensory conditions, yielding scores from 0 (complete imbalance) to 100 (no sway) (20). For clarity, the variables analyzed include “visual”, representing overall performance in the visual sensory condition; “visual dependence”, reflecting the extent to which postural stability is influenced by visual input; and “visual preference”, referring to the patient's reliance on visual cues over vestibular ones. In these cases, the brain learns to rely more on physical sensations than on vision, which can mask the underlying balance issue (20). Using these six measured conditions, a calculation was performed to analyze their balance with respect to somatosensory, visual, and vestibular coefficients, as well as the global balance parameter (21) and the number of falls (≥ 3 falls indicated a pathological fall pattern) were determined (21). Additionally, fall history was assessed on the first visit using a three-question survey (22).

The assessment of CRF included a peak, symptom-limited cardiopulmonary exercise test performed on an electronically braked Lode Excalibur Sport Cycle Ergometer (Groningen, the Netherlands). The ramp protocol began at 40 W (∼70 rpm) with a gradual 10 W increment every minute in the ramp protocol. A continuous electrocardiogram was registered throughout the test, and blood pressure was recorded every two minutes. Expired gas was analysed with a system (USM001 V1.0, Ergo CardMedi-soft, Belgium) calibrated before each test to determine the first ventilatory threshold (VT_1_) and peak oxygen uptake (V˙O_2peak_). A valid V˙O_2peak_ test will be defined by the fulfilment of at least two of the following four criteria: attainment of 85% of the age-predicted maximum heart rate, a rating of perceived exertion >18 on th Borg scale, a peak respiratory exchange ratio ≥ 1.10, or the absence of any further increase in oxygen uptake or heart rate despite a progressive increase in exercise workload (23). Furthermore, maximum effort was confirmed by exhaustion, defined as the inability to maintain the required pedaling cadence despite vigorous verbal encouragement during the final minutes of the test.

The HRQoL was assessed using the 25-item validated Dizziness Handicap Inventory (DHI) questionnaire, which evaluated physical (7 items), functional (9 items), and emotional (9 items) aspects, with scores ranging from 0 to 4 per question (“yes” = 4 points, “sometimes” = 2 points, “no” = 0 points). Scores indicated *standard* handicap (0–14), *mild* (16–34), *moderate* (36–52), and *severe* (54+) quality of life impact (24). The emotional profile was evaluated using the Beck Depression Inventory (BDI-II) and the Beck Anxiety Inventory (BAI), both 21-item self-report questionnaires with scores ranging from 0 to 63. BDI-II cut-offs were 0–13 for *minimal*, 14–19 for *mild*, 20–28 for *moderate*, and 29–63 for *severe*, and BAI scores were classified as *minimal* (0–7), *mild* (8–15), *moderate* (16–25), and *severe* anxiety (26–63) (25, 26).

Physical activity and sedentary behaviour were assessed via the short-form International Physical Activity Questionnaire (IPAQ) (27) to evaluate *vigorous* PA (VPA), *moderate* PA (MPA), and time spent walking and sitting (28). For analysing sleep quality, each participant wore a triaxial accelerometer (GT3X + ActiGraph, Pensacola, Florida, USA) on their non-dominant wrist for seven consecutive days (except during water activities), attached with a Velcro strap. Eligibility for analysis required a minimum of three days of device wear, including one weekend day. All participants exceeded this wear-time criterion (29). No other devices were implemented during this period. Sleep was measured using Actilife 6.11.9 (60-s epochs) on downloaded ActiGraph data. Extracted sleep variables included the total sleep time (TST) (i.e., minutes of sleep between sleep onset and wake time), WASO (i.e., total minutes awake once they fall asleep), total bedtime, and sleep efficiency (< 85% indicated *low* efficiency), which was obtained by calculating ([total sleep time/total bedtime] × 100) (30). A validated algorithm based on the Kripke scoring method (31) assessed the raw ActiGraph data to calculate sleep time. Parameters for PA were also obtained, including the total PA and sedentary time.

### 2.3 Statistical analysis

Data normality was assessed using the Shapiro–Wilk test. Variables were expressed as mean ± standard deviation or median (IQR), as appropriate. Sex differences were analyzed using independent t-tests or Mann–Whitney U tests. Chi-square tests assessed categorical variables. Effect sizes were interpreted as follows: Cohen's *d*, trivial (<0.20), small (0.20 to <0.50), moderate (0.50 to <0.80), and large (≥ 0.80); eta-squared (*η*_2_), trivial (<0.01), small (0.01 to <0.06), moderate (0.06 to <0.14), and large (≥ 0.14) and Phi coefficient (*φ*), trivial (<0.10), small (0.10 to <0.30), moderate (0.30 to <0.50), and large (≥ 0.50) (32). Statistical significance was set at *P* < 0.05. Missing data occurred only when participants did not complete the required assessments; thus, a complete-case analysis approach was employed. Given the descriptive and exploratory nature of this study and the number of outcome variables analyzed, no multiple-comparison adjustment was applied. Therefore, results with borderline significance (*P* values close to 0.05) were interpreted with caution as hypothesis-generating findings.

## 3 Results

### 3.1 Sex-specific characteristics

**Figure 1:**
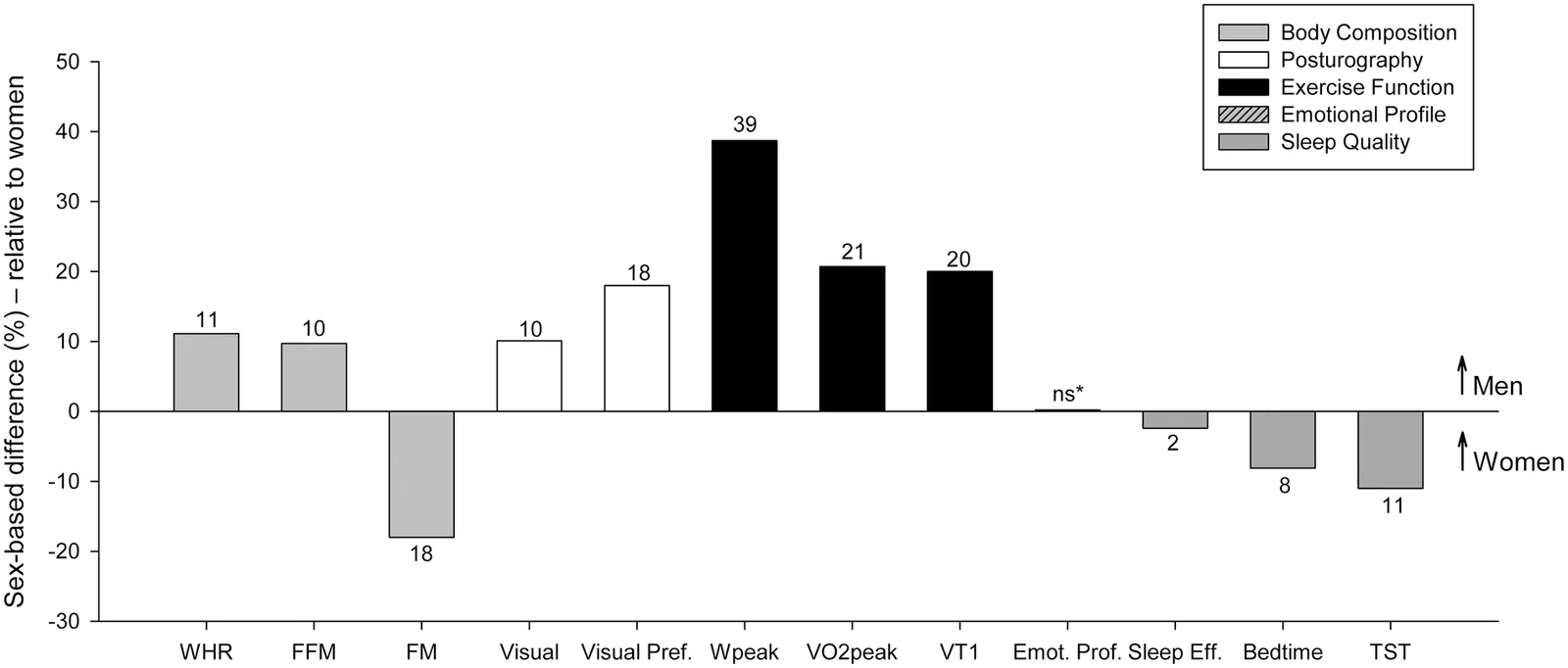
Sex-specific differences in people with chronic vestibular hypofunction. Grey colums represent body composition and sleep quality; white colums represent equilibrium and sensory organisation measured by posturography; black colums exercise function; and the column with diagonal lines represents emotional profile. Emot. Prof. (emotional profile), FM (fat mass), FFM (fat-free mass), Visual Pref. (visual preference), Sleep Eff. (sleep efficiency), TST (total sleep time), VO2peak (peak oxygen uptake), VT1 (first ventilatory threshold), WHR (waist-to-hip ratio), Wpeak (peak load on the cardiorespiratory exercise test).

Participants showed a mean WHR of 0.8 (IQR = 0.7, 0.9). Significant sex differences were observed for age, body mass, waist circumference, WHR, FFM, FM, and resting DBP. Men showed higher body mass, WHR, FFM, and DBP, with ES ranging from *moderate* to *large*, while women showed higher FM, with a *moderate* ES (Table 1; Figure 1).

**Table 1:** Body composition, resting haemodynamic variables, medication use, smoking status, and diabetes mellitus profiles of the EXERVEST participants. Values are mean ± standard deviation, median (interquartile range) or percentage (%).

Variables	All (*N* = 67)	Women (*N* = 43)	Men (*N* = 24)	*P* W vs. M	*d Cohen* */* *Eta squared (η^2^)/ φ*	Effect size interpretation
Age (yrs)	58.0 (52.0, 66.0)	59.0 (54.0, 67.0)	54.5 (47.8, 61.8)	0.019*	0.081	*Moderate*
Total body mass (kg)	70.5 (59.5, 85.6)	64.0 (55.9, 77.9)	85.3 (72.2, 102.2)	<.001*	0.296	*Large*
Waist (cm)	88.2 ± 14.4	83.0 ± 12.1	97.6 ± 13.9	<.001*	1.140	*Large*
WHR (Waist/Hip)	0.8 (0.7, 0.9)	0.8 (0.7, 0.9)	0.9 (0.8, 1.0)	0.002*	0.139	*Large*
FFM (%)	67.2 ± 9.0	64.9 ± 8.2	71.2 ± 9.2	0.006*	0.730	*Moderate*
FM (%)	32.8 ± 9.0	35.1 ± 8.2	28.8 ± 9.2	0.006*	0.730	*Moderate*
Rest SBP (mmHg)	119.9 ± 12.7	117.9 ± 12.5	123.6 ± 12.4	0.074	0.463	*Small*
Rest DBP (mmHg)	77.3 ± 9.5	75.0 ± 8.2	81.4 ± 10.5	0.008*	0.703	*Moderate*
Rest HR (bpm)	70.2 ± 8.0	70.9 ± 6.3	69.0 ± 10.5	0.434	0.232	*Small*
MEDICATION						
ACEI (*n*, %)	9, 13.4	5, 11.6	4, 16.7	0.588	0.067	*Trivial*
ARB (*n*, %)	7, 10.5	6, 14.0	1, 4.2	0.199	0.158	*Small*
Diuretic (*n*, %)	4, 6.0	4, 9.3	0, 0.0	0.119	0.192	*Small*
Statin (*n*, %)	13, 19.4	7, 16.3	6, 25.0	0.413	0.101	*Small*
Hypoglycemic agent (*n*, %)	5, 7.5	2, 4.7	3, 12.5	0.253	0.141	*Small*
Antithrombotic (*n*, %)	12, 17.9	7, 16.3	5, 20.8	0.673	0.052	*Trivial*
Antidepressant (*n*, %)	3, 4.5	2, 4.7	1, 4.2	0.911	0.014	*Trivial*
Bronchodilator (*n*, %)	6, 9.0	5, 11.6	1, 4.2	0.293	0.129	*Small*
Thyroid hormone (*n*, %)	7, 10.5	6, 14.0	1, 4.2	0.199	0.158	*Small*
BB (*n*, %)	2, 3.0	1, 2.3	1, 4.2	0.684	0.050	*Trivial*
Cigarette smoking (*n*, %)	12, 17.9	6, 14.0	6, 25.0	0.278	0.134	*Small*
Diabetes Mellitus (*n*, %)	6, 9.0	2, 4.7	4, 16.7	0.106	0.199	*Small*

ACEI, angiotensin-converting-enzyme inhibitors; ARB, angiotensin II receptor blockers; BB, beta-blockers; DBP, diastolic blood pressure; FM, fat mass; FFM, fat-free mass; HR, heart rate; M, men; N, sample size; SBP, systolic blood pressure; W, women; WHR, waist-to-hip ratio.

*Significant difference between women and men was set at *P* < 0.05.

More than half of the participants (56.7%) were taking medication. Antihypertensives, statins, hypoglycaemic agents, bronchodilators, antidepressants, and thyroid hormones were commonly prescribed. Smoking prevalence was 17.9%.

### 3.2 Physical, functional, and physiological outcomes

All participants obtained a mean total score of 61.0 (IQR = 57.5, 63.0) points in the MDGI regarding balance and fall risk (Table 2), with no significant differences between sexes (*P* > 0.05). Overall, participants showed values above the 75th percentile in somatosensory (88.0 [IQR = 74.8, 97.0]), visual (78.0 [IQR = 70.5, 87.3]), and visual preference (79.0 [IQR = 58.0, 94.0]) in the dynamic posturography, using the age-matched healthy normative database provided by the Synapsys Posturography System as a reference (19). However, the vestibular value (49.5 [IQR = 29.8, 74.3] %) was near the 50th percentile. Visual and visual preference both displayed differences between sexes (Table 2), with men obtaining significantly higher vision dependence results than women in these two variables (*η^2^* = *moderate*, Figure 1). Both sexes showed the same risk of falls (women and men: 1.0 [IQR = 0.0, 2.0] point).

**Table 2:** Participants’ equilibrium measured by the dynamic posturography sensory organisation test and MDGI. Values are mean ± standard deviation or median (interquartile range).

Variables	All (*N* = 66)	Women (*N* = 42)	Men (*N* = 24)	*P* W vs. M	*d Cohen* */* *Eta squared (η^2^)*	Effect size interpretation
MDGI (points)	61.0 (57.5, 63.0)	60.0 (56.0, 62.0)	63.0 (58.0, 64.0)	0.078	0.048	*Small*
POSTUROGRAPHY						
Global (%)	49.9 ± 15.9	47.2 ± 16.4	54.5 ± 14.1	0.072	0.453	*Small*
Somatosensory (%)	88.0 (74.8, 97.0)	84.5 (67.0, 96.3)	94.0 (76.0, 97.8)	0.080	0.046	*Small*
Visual (%)	78.0 (70.5, 87.3)	75.5 (66.8, 84.3)	84.0 (76.3, 89.5)	0.025*	0.076	*Moderate*
Vestibular (%)	49.5 (29.8, 74.3)	50.5 (25.3, 76.8)	47.5 (42.5, 72.5)	0.915	0.000	*Trivial*
Visual Preference (%)	79.0 (58.0, 94.0)	73.0 (52.0, 88.0)	89.0 (73.5, 94.8)	0.013*	0.094	*Moderate*
Fall Risk (points)	1.0 (0.0, 2.0)	1.0 (0.0, 2.0)	1.0 (0.0, 2.0)	0.416	0.010	*Small*

M, men; MDGI, modified dynamic gait index; N, sample size; W, women.

*Significant difference between women and men was set at *P* < 0.05.

Participants achieved maximum exercise effort (RER = 1.1 ± 0.1). Women demonstrated lower workload, exercise time, distance, peak SBP, absolute and relative V˙O_2peak_, V˙CO_2peak_, and VT1 than men, showing *moderate* to *large* magnitude differences. The median relative V˙O_2peak_ was 21.0 (IQR = 18.0, 25.0 mL·kg^−1^·min^−1^) in women and 26.5 (IQR = 20.3, 31.0 mL·kg^−1^·min^−1^) in men (Table 3; Figure 1).

**Table 3:** Participants’ exercise function. Values are mean ± standard deviation or median (interquartile range).

Variables	All (*N* = 67)	Women (*N* = 43)	Men (*N* = 24)	*P* W vs. M	*d Cohen* */* *Eta squared (η^2^)*	Effect size interpretation
Workload_peak_ (W)	115.0 (82.5, 157.8)	98.0 (75.8, 118.3)	160.0 (132.8, 192.0)	<.001*	0.379	*Large*
Time_peak_ (min)	9.0 (6.0, 13.0)	7.0 (5.0, 9.0)	13.0 (11.3, 16.5)	<.001*	0.388	*Large*
Distance_peak_ (km)	1.3 (0.8, 2.3)	1.0 (0.7, 1.4)	2.4 (2.0, 3.4)	<.001*	0.407	*Large*
SBP_peak_ (mmHg)	198.8 ± 30.5	192.6 ± 29.4	209.2 ± 30.1	0.033*	0.559	*Moderate*
DBP_peak_ (mmHg)	104.0 (91.5, 113.0)	106.0 (92.0, 114.0)	100.0 (89.0, 113.0)	0.714	0.002	*Trivial*
HR_peak_ (bpm)	159.0 (144.0, 168.0)	159.0 (141.0, 166.0)	164.0 (151.8, 173.5)	0.169	0.028	*Small*
V̇O_2peak_ (L·min·^−1^)	1.6 (1.3, 2.0)	1.3 (1.2, 1.6)	2.0 (1.8, 2.6)	<.001*	0.474	*Large*
V̇O_2peak_ (mL·kg^−1^·min^−1^)	21.0 (19.0, 28.0)	21.0 (18.0, 25.0)	26.5 (20.3, 31.0)	0.009*	0.103	*Moderate*
V̇CO_2peak_ (L·min^−1^)	1.7 (1.3, 2.1)	1.5 (1.2, 1.8)	2.2 (1.8, 2.7)	<.001*	0.410	*Large*
RER_peak_	1.1 ± 0.1	1.1 ± 0.1	1.1 ± 0.1	0.136	0.385	*Moderate*
MET_peak_	6.0 (5.4, 8.0)	6.0 (5.1, 7.1)	7.6 (5.8, 8.9)	0.009*	0.103	*Moderate*
VT_1_ (mL·kg^−1^·min^−1^)	13.0 (11.0, 16.0)	12.0 (10.0, 15.0)	15.0 (11.3, 18.5)	0.038*	0.071	*Moderate*

DBP_peak_, peak diastolic blood pressure; HR_peak_, peak heart rate; M, men; MET_peak_, peak metabolic equivalent of task; N, sample size; RER_peak_, peak respiratory exchange ratio; SBP_peak_, peak systolic blood pressure; V̇CO_2peak_, peak carbon dioxide production; V̇O_2peak_, peak oxygen uptake; VT_1_, first ventilatory threshold, W, women.

*Significant difference between women and men was set at *P* < 0.05.

### 3.3 Emotional outcomes

According to HRQoL and emotional profile variables, the median DHI total score was 26.9, ranging from 16 to 34 points, with the emotional subscale revealing the lowest median score of 5.0 (IQR = 2.0, 9.0) out of a maximum of 36 points. The median BDI-II and BAI scores were 11.0 (IQR = 4.0, 17.5), and 12.0 (IQR = 7.8, 20.3), respectively. No significant sex-based differences were found in the physical, functional, and emotional profiles assessed using the DHI, BDI-II, and BAI questionnaires (Table 4).

**Table 4:** Participants’ health-related quality of life and emotional profile. Values are mean ± standard deviation or median (interquartile range).

Variables	All (*N* = 65)	Women (*N* = 42)	Men (*N* = 23)	*P* W vs. M	*d Cohen* */* *Eta squared (η^2^)*	Effect size interpretation
DHI (total)	22.0 ± 11.2	21.4 ± 10.9	23.0 ± 12.0	0.598	0.120	*Trivial*
DHI (physical)	8.1 ± 3.5	7.9 ± 3.6	8.4 ± 3.3	0.646	0.138	*Trivial*
DHI (emotional)	5.0 (2.0, 9.0)	5.5 (2.0, 9.0)	5.0 (2.0, 11.0)	0.397	0.011	*Small*
DHI (functional)	7.0 (4.0, 11.0)	7.0 (4.8, 11.0)	7.0 (3.0, 12.0)	0.945	0.000	*Trivial*
BDI-II	11.0 (4.0, 17.5)	10.0 (5.0, 17.5)	12.0 (2.0, 17.5)	0.259	0.020	*Small*
BAI	12.0 (7.8, 20.3)	11.5 (7.8, 20.0)	13.5 (7.3, 29.3)	0.496	0.007	*Trivial*

BAI, Beck Anxiety Inventory; BDI-II, Beck Depression Inventory-II; DHI, Dizziness Handicap Inventory; M, men; N, sample size; W, women.

### 3.4 PA patterns and sleep quality outcomes

As regards participants’ PA level and sedentary behaviour (Table 5), both IPAQ (2152.5 ± 1067.2 min/week) and accelerometer-based (480.3 [IQR = 420.3, 572.1] min/day) data indicated that participants spent more sedentary time than in total PA (425.5 ± 115.6 min/day). No other sex-based differences (*P* > 0.05) were found in PA and sedentary behaviour variables.

**Table 5:** Physical activity levels and sleep quality of study population. Values are mean ± standard deviation or median (interquartile range).

Variables	All (*N* = 65)	Women (*N* = 42)	Men (*N* = 23)	*P* W vs. M	*d Cohen* */* *Eta squared (η^2^)*	Effect size interpretation
IPAQ						
VPA (min/week)	0.0 (0.0, 0.0)	0.0 (0.0, 0.0)	0.0 (0.0, 0.0)	0.066	0.063	*Moderate*
MPA (min/week)	0.0 (0.0, 138.8)	0.0 (0.0, 142.5)	0.0 (0.0, 210.0)	0.874	0.000	*Trivial*
Walking (min/week)	275.0 (140.0, 420.0)	240.0 (165.0, 420.0)	315.0 (123.8, 630.0)	0.232	0.026	*Small*
Sitting (min/week)	2152.5 ± 1067.2	2132.1 ± 1114.3	2193.3 ± 996.0	0.845	0.057	*Trivial*
ACCELEROMETER						
Physical activity						
Sedentary time (min/day)	480.3 (420.3, 572.1)	461.4 (336.3, 550.3)	485.9 (455.6, 571.4)	0.337	0.014	*Small*
TPA (min/day)	425.5 ± 115.6	429.6 ± 124.0	418.0 ± 100.4	0.704	0.099	*Trivial*
Sedentary time (%)	54.4 ± 10.7	53.4 ± 11.9	56.3 ± 8.1	0.302	0.270	*Small*
TPA (%)	45.6 ± 10.7	46.6 ± 11.9	43.7 ± 8.1	0.302	0.270	*Small*
Sleep						
Sleep Efficiency (%)	92.0 ± 3.5	92.8 ± 2.9	90.6 ± 4.0	0.014*	0.654	*Moderate*
Bedtime (min/day)	447.3 (416.7, 475.1)	455.3 (418.4, 475.7)	429.2 (381.6, 459.9)	0.022*	0.081	*Moderate*
TST (min/day)	406.8 (378.5, 442.8)	422.0 (391.3, 449.0)	390.7 (338.8, 425.3)	0.011*	0.099	*Large*
WASO (min/day)	35.7 ± 14.7	33.5 ± 13.8	39.7 ± 15.6	0.107	0.424	*Small*

IPAQ, International Physical Activity Questionnaire-short form; M, men; MPA, moderate physical activity; N, sample size; TPA, total physical activity; sleep efficiency (%), total sleep time divided by total bedtime multiplied by 100; TST, total sleep time; VPA, vigorous physical activity; W, women; WASO, wake after sleep onset.

*Significant difference between women and men was set at *P* < 0.05.

The sleep analysis from the accelerometer (Table 5) revealed a mean sleep efficiency above 85%, with a TST of 6.8 h and WASO above 30 min, indicating frequent awakenings during the night. There were significant sex differences in sleep efficiency (*P* = 0.014, *d* *=* *moderate*), bedtime (*P* = 0.022, *η^2^* = *moderate*), and TST (*P* = 0.011, *η^2^* = *large*), with women demonstrating higher values for these sleep parameters than men (Figure 1). These effect sizes indicate a moderate-to-large clinical relevance in sleep duration and quality between sexes.

## 4 Discussion

This cross-sectional study provides a comprehensive characterization of the physical, functional, physiological, and emotional profile of adults diagnosed with CVH, with a particular focus on sex-related differences, within the EXERVEST study. Overall, 1) participants showed a relatively low fall risk and preserved somatosensory and visual contributions to postural control, *minimal* depression symptoms and *optimal* sleep efficiency. However, several unfavourable findings coexisted, including borderline abdominal obesity, impaired vestibular posturography performance, *mild* HRQoL impairment and *mild* anxiety symptoms, high sedentary behaviour, low self-reported PA, short sleep duration, and elevated WASO, and 2) women and men differed on several physical, functional, and physiological outcomes (Figure 1). Given the cross-sectional nature of the study, these findings should be interpreted as associations rather than evidence of causal relationships.

The assessed participants showed a body fat percentage (FM = 32.8 ± 9.0%) and waist circumference values (83.0 ± 12.1 cm in women and 97.6 ± 13.9 cm in men) that fell close to established thresholds for cardiometabolic risk (33). In the absence of a healthy control group, this should be interpreted relative to published reference ranges rather than as evidence of a typical obesity profile in people with CVH. Preclinical work in high-fat-diet mice has proposed that vestibular lesions and hyperinsulinaemia alter gene expression in the vestibular nuclei through changes in the vestibular sensory epithelium, suggesting a possible bidirectional link between vestibular and metabolic dysfunction (34), this mechanism, however, has not been confirmed in humans. Consistent with this caveat, a recent observational and Mendelian randomization analysis did not find a robust causal relationship between metabolic syndrome and vestibular dysfunction, even though low high-density lipoprotein-cholesterol and hypertension emerged as potential risk factors (35). Taken together, the mechanistic link between obesity, glucose metabolism, and vestibular impairment remains hypothetical rather than established, and the proportion of participants in our sample with diagnostic diabetes mellitus (9%), hypoglycaemic agent use (7.5%), and statins pharmacology (19.4%) (Table 1) should be regarded as a descriptive feature of this cohort rather than support for this pathway.

Previous studies have confirmed that people with CVH engage in less moderate-to-vigorous PA than healthy adults (3, 16). In the present study, participants’ self-reported PA levels, as measured by the IPAQ questionnaire, indicated no moderate-to-vigorous PA, only light-intensity walking (275 min/week), and high sedentary time (2152.5 ± 1067.2 min/week). These findings were further supported by accelerometer data, which showed that sedentary time (54.4%) exceeded time in motion (45.6%) (Table 5), yet recorded a substantially higher volume of total daily PA (425.5 ± 115.6 min/day) than would be inferred from the IPAQ alone. This divergence is consistent with the well-documented tendency of self-report questionnaires to under- or overestimate activity relative to device-based measures (36, 37), and may partly reflect that much of participants’ movement occurred at a light intensity not captured by the IPAQ's moderate-to-vigorous categories. The condition itself, and specifically their subjective feeling of insecurity when walking and the potential risk of falling reported in CVH (3), may contribute to avoidance behaviours and a more sedentary lifestyle, which could plausibly reinforce postural instability and general deconditioning over time; however, the cross-sectional design of this study cannot establish the direction of this relationship. These findings, nonetheless, reinforce the importance of promoting PA and healthy lifestyle behaviours in people with CVH.

There is growing evidence suggesting that sex differences play a crucial role in the clinical presentation of various diseases. In our sample, men and women differed in FFM and FM (Table 1), with both groups close to established thresholds for high body fat percentage (i.e., ≥ 25% for men and ≥ 35% for women) (38). Women were, however, significantly older than men (59.0 vs. 54.5 years old, *P* = 0.019), and age is itself an established determinant of body composition and fat distribution, because age was not adjusted fo in these comparisons, the extent to which the observed differences reflect sex, age, or their interaction cannot be determined from the present data, and this should temper any mechanistic interpretation. Hormonal modulation and anatomical differences in vestibular structures have been proposed in the literature as possible contributors to the higher prevalence of CVH among women (14). As these factors were not measured in EXERVEST, they should be regarded as hypotheses drawn from previous research rather than explanations supported by our findings.

The MDGI results (Table 2) indicated a low risk of falls in this population. However, after analyzing dynamic posturography data focusing on the three central afferent sensory systems for maintaining postural control (visual, vestibular, and proprioceptive), the average global (49.9 ± 15.9%) and vestibular (49.5 [IQR = 29.8, 74.3] %) variables were vastly reduced, showing a balance impairment and instability when postural defence mechanisms are stressed to the limits (19). Women showed lower values than men in the visual (−10.1%) and visual preference (−18.0%) variables, meaning that, on average, they relied comparatively less on visual input to maintain balance. Possible explanations proposed in the literature include a sexually dimorphic vestibular system with anatomical differences between females and males (14), and reduced oestrogen and progesterone levels in post-menopausal women, which have been linked to inner-ear microcirculation disorders (12). Although in the present study 41.9% of the women were in this life stage, these mechanisms were not directly assessed and should be regarded as plausible, literature-based hypotheses rather than explanations confirmed by our data.

Regarding the exercise function, women showed lower CRF values than men (21.0 vs. 26.5 mL·kg^−1^·min^−1^), together with lower workload (98.0 vs. 160.0 W), total exercise time (7.0 vs. 13.0 min), covered distance (1.0 vs. 2.4 km), and VT_1_ (12.0 vs. 15.0 mL·kg^−1^·min^−1^). Despite consistently lower measured values, women's relative V˙O_2peak_ corresponded to a higher percentile ranking than men's when benchmarked against sex-specific normative tables (i.e., “excellent” vs. “fair”) (33, 39). This apparent paradox arises because CRF reference values are stratified by sex (i.e., a woman's score is compared only with other women's scores, so the resulting percentile reflects standing relative to same-sex peers rather than absolute physiological capacity). It should therefore be made explicit that these categories do not indicate that women had a higher exercise capacity than men in absolute terms; on the contrary, every direct performance marker was lower in women, a pattern that women's older mean age may partly influence in this sample. This disparity is consistent with long-established physiological principles compared with men, since women generally have a smaller heart, a lower blood volume, and a reduced blood oxygen-carrying capacity, with corresponding lower cardiac filling and stroke volume, all of which constrain oxygen delivery during exercise. Experimental work that matched blood volume and oxygen-carrying capacity between sexes found that V˙O_2peak_ gap was fully abolished once these blood attributes were equalized, indicating that a substantial part of the sex difference in CRF is explained by these haematological factors rather than by sex *per se* (40) (Table 3). International normative classifications should therefore be interpreted with caution in this population as they reflect relative standing within each sex rather than an absolute physiological advantage.

Current clinical data suggest that the cerebellum and vestibular system are involved in emotional processing, with an internal hypothesis receiving erroneous information that could, in turn, generate an alteration in sensory integration and contribute to anxiety and depression (4). Indeed, for many years, people with CVH have reported experiencing elevated depression and anxiety, particularly in the elderly and with more advanced condition (41), a pattern reported for both sexes, suggesting that mental-health symptoms are common in CVH (42). Our participants reported *mild* handicaps related to HRQoL, *minimal* depression, and *mild* anxiety, with no differences between sexes (Table 4). These relatively favourable questionnaire-based results should be interpreted cautiously because only self-report instruments were used; therefore, our data cannot determine whether participants’ perception of imbalance was free of psychological influence, nor whether the condition genuinely spared their emotional wellbeing in daily life. A previous study confirmed that psychological distress experienced by people with CVH is broader than registered with questionnaires and may not give an accurate view of symptoms and their consequences (5). Therefore, a mixed-methods approach combining qualitative and quantitative reports would be of interest to clarify the bidirectional relationship between neuro-otologic conditions and psychological disorders. Based on previous literature rather than on the present findings alone, it has been suggested that psychiatric disorders should be considered part of the differential diagnosis in participants with CVH (4, 41, 42). Results from the present study, showing *mild* anxiety and *minimal* depression scores, do not contradict this recommendation but cannot independently confirm it.

A significant association has been established between poor sleep and a broad spectrum of psychological and physiological impairments in the CVH population (6). This relationship between dizziness and sleep is reciprocal, underscoring its complexity (8). In the present study, women exhibited a daily average sleep duration of 455.3 (IQR = 418.4, 475.7) minutes (7.6 h), approximately 26 min longer than men (429.2 [IQR = 381.6, 459.9] min = 7.2 h, *P* = 0.022). While statistically significant, a difference of this magnitude is modest in absolute terms, falls within the range of ordinary night-to-night variability for a given individual, and its meaningfulness at a population level would benefit from confirmation in a larger, more balanced sample and from correlation with daytime functioning or symptom severity. According to the general recommendations of the National Sleep Foundation, the leading indicators of good sleep quality are sleep efficiency and WASO (43). In the current investigation, both women and men demonstrated outstanding sleep efficiency, with a TST-to-bedtime ratio exceeding 85% (i.e., TST divided by bedtime) (30). However, given that a WASO of 41 min or more is not considered indicative of good sleep quality (43), this finding could be questioned in the CVH population (women: 33.5 ± 13.8 min/day, men: 39.7 ± 15.6 min/day) and could negatively impact overall wellbeing and cognitive domains (44).

Overall, these results emphasise the need for guidance and support to help people with CVH adopt healthy behaviours, such as regular PA, to cope better with vestibular symptoms and reduce functional disability. While our study identified several differences between women and men with CVH, it is also important to acknowledge certain limitations when interpreting these findings. First, the cross-sectional and descriptive design does not allow causal inferences about the relationships between CVH and the physical, functional, or emotional variables assessed, nor about the direction of the associations discussed above; longitudinal or interventional studies are needed to clarify these relationships. Second, no healthy control group was included, so the observed profile cannot be concluded to be specific to, or caused by, CVH rather than reflecting general characteristics of a middle-aged to older adult population; comparisons were instead made against published reference values and normative databases. Third, women were significantly older than men, and age was not adjusted for in the sex-based comparisons. Because age can independently influence body composition, CRF, balance, and sleep, some of the reported sex differences may be partly confounded by age, which should temper mechanistic interpretations attributed solely to sex. Fourth, a large number of independent statistical tests were performed across five outcome domains at a conventional threshold of *P* < 0.05 without correction for multiplicity; findings with small-to-moderate effect sizes are the most susceptible to being false positives, and false discovery rate control offers a less conservative alternative to Bonferroni-type correction that could be applied in future analyses of this data set (45). Fifth, the sample combined heterogeneous aetiologies of CVH, with disease duration ranging from 1 to 44 years, but we ensured that participants were not taking any medication for vertigo to minimize potential interference. Furthermore, unilateral and bilateral presentations in particular are known to differ in clinical subtype, symptom burden, and HRQoL (46, 47), so pooling these subgroups may have masked clinically relevant differences. Sixth, the periods of vertigo/dizziness experienced by participants with some subpathologies made recruitment challenging, which together with the complexity of diagnosing CVH, likely contributed to the modest and unequal sex distribution in our sample (35.8% men) and limited statistical power for some between-sex comparisons. Finally, more than half of the participants (56.7%) were taking medication for cardiovascular, metabolic, or psychiatric conditions, which may have independently influenced body composition, blood pressure, mood, or sleep outcomes.

## 5 Conclusions

Adults with CVH exhibited a multidimensional clinical profile characterized by impairments extending beyond vestibular-mediated balance to include physical, behavioural, physiological, and emotional domains. Women and men differed in body composition, balance performance, CRF, and sleep duration. Still, these findings should be interpreted cautiously given the cross-sectional design and potential confounding factors. These findings support the value of a comprehensive clinical assessment of individuals with CVH and warrant further longitudinal research to determine the clinical relevance of sex-related differences and their implications for personalized programs.

## Data Availability

Data are available on reasonable request from the corresponding author.

## Supplementary material

Supplementary File 1 Characterisation of chronic vestibular hypofunction. Distinct physical, functional, physiological, and emotional variables are categorised based on their clinical presentation into positive (green) and negative (red) markers. For sexual dimorphism, statistical significance was set up at *P* < 0.05.
