# Supplementary material for: Sex-related differences in the physical, functional, physiological, and emotional profile of adults with chronic vestibular hypofunction: a cross-sectional EXERVEST study
Source: Front Rehabil Sci. 2026 Aug 26;7:1890080. doi: 10.3389/fresc.2026.1890080 (PMC13555604; doi:10.3389/fresc.2026.1890080)

Vestibular Hypofunction's Profile

Positive vs. Negative Markers

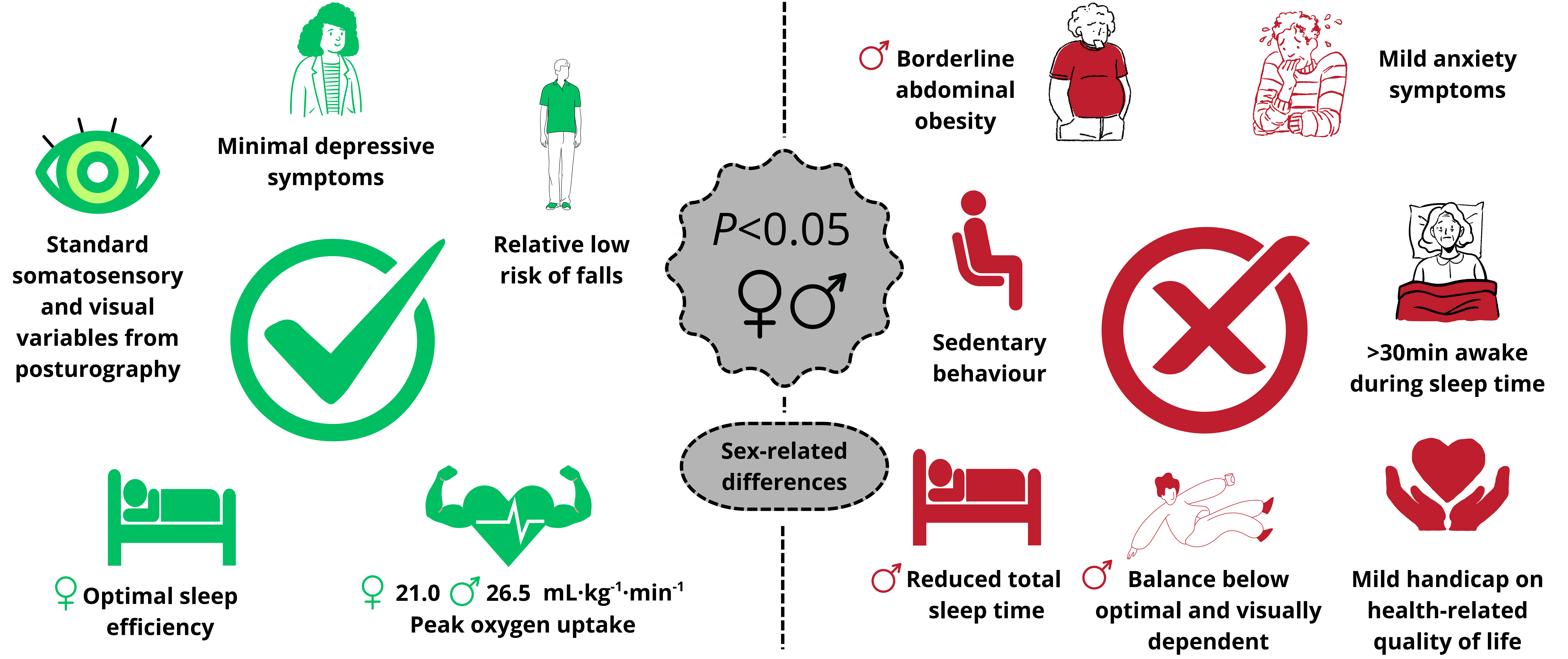

Supplement: Supplementary file 1 [file Image1.pdf]
